# Effect of Bacterial Resistance of *Escherichia coli* From Swine in Large-Scale Pig Farms in Beijing

**DOI:** 10.3389/fmicb.2022.820833

**Published:** 2022-03-31

**Authors:** Xiaoxia Liu, Qian Liu, Yongyou Cheng, Rui Liu, Ruting Zhao, Jishi Wang, Yanyun Wang, Shuming Yang, Ailiang Chen

**Affiliations:** ^1^Key Laboratory of Agro-Product Quality and Safety, Institute of Quality Standards and Testing Technology for Agro-Products, Chinese Academy of Agricultural Sciences, Beijing, China; ^2^Department of Food Engineering, Light Industry Polytechnic College, Beijing, China; ^3^School of Investigation, People’s Public Security University of China, Beijing, China

**Keywords:** pig farm, *Escherichia coli*, multi-drug resistance, factor, antibiotic resistance

## Abstract

With widespread use of antibiotics in the aquaculture industry, bacterial resistance has recently attracted increasing attention. Continuous emergence of multi-resistant bacteria has greatly threatened human and animal health, as well as the quality and safety of livestock products. To control bacterial resistance, the effect of bacterial resistance needs to be well understood. The purpose of this study was to explore the factors influencing *Escherichia coli* (*E. coli*) drug resistance in large-scale pig farms. In this study, 296 strains of *E. coli* isolated and identified from large-scale pig farms in Beijing were used as the research objects. *In vitro* drug sensitivity tests were used to determine the sensitivity to 10 antibiotics of pig-derived *E. coli*. SPSS logistic regression was employed to analyze the effects of the season, pig type, sampling point (medication type) and sampling location on resistance and multi-drug resistance of *E. coli* from pigs. The degrees of drug resistance to 10 antibiotics of the 296 strains of pig-derived *E. coli* were varied, their resistance rates were between 4.05 and 97.64%, and their multi-drug resistance was appalling, with the highest resistance to six antibiotics being 26.35%. The isolated strains were proven more resistant to tetracyclines, penicillin and chloramphenicol, which are commonly used for disease prevention in pig farms, and less resistant to quinolones and aminoglycosides, which are not used in pig farms. The resistance of the isolated strains in spring and summer was generally higher than that in winter. *E. coli* resistance in piglets, fattening pigs and sows was more serious than that in nursery and sick pigs. The results showed that the season, type of medication and type of pig had an influence on the pig-derived *E. coli* resistance, among which the type of medication was the most influencing factor.

## Introduction

Antibiotic resistance (AR) affects treatment of bacterial diseases in humans and animals and will become one of the major threats facing the world in the future (World Health Organization, 2012; Centner, 2016; Tornimbene et al., 2018; Borgmann et al., 2020). The main reasons for bacterial resistance development are the abuse of antibiotics and scarce measures to control the spread of resistant bacteria. Antibiotic use generates selective pressure that is conducive to resistant bacteria development and allows the bacteria to spread among bacterial populations in humans, animals and the environment (Baquero, 2011; World Health Organization, 2012). The research showed that the number of deaths due to drug-resistant bacterial diseases was estimated to be 50,000 each year in Europe and the United States. By 2050, it is estimated that the number of such deaths in the world will reach 10 million each year, posing a threat to global economy and biosecurity (Zhang et al., 2017; Courtenay et al., 2019). In the aquaculture industry and others, antibiotics are employed to promote animal growth and to prevent or treat diseases (Cabello, 2006; Sarmah et al., 2006; Kuang et al., 2020). However, due to antibiotic abuse, bacteria have increased occurrence of drug resistance as they increased selective pressure and generated drug resistance pools (van den Bogaard, 2000; Smith et al., 2002), which not only diminished treatment options for farmers, but also led to harder-to-treat animal diseases resulted from multidrug-resistant (MDR) bacteria (Ramchandani et al., 2005; Michael et al., 2012). Magiorakos et al. (2012) have claimed that enterobacteriaceae strains are MDR if they are non-susceptible to more than one agent in over 3 antimicrobial categories, extensively drug-resistant (XDR) if they are non-susceptible to more than one agent in all but over 2 categories and pandrug-resistant (PDR) if they are non-susceptible to all the listed antimicrobial agents.

Swine production accounts for a large proportion of global meat production (Osterberg et al., 2016), and the use of antibiotics in food animals may lead to the spread of drug-resistant bacteria from livestock to human through the food chain (Meemken et al., 2008; Chantziaras et al., 2014). *Escherichia coli* (*E. coli*) is an intestinal symbiotic bacterium that exists in animals and humans and is susceptible to high selection pressure from antimicrobial agents in contact with the host. Therefore, *E. coli* is often used as a carrier for monitoring antimicrobial resistance in human or livestock groups (European Food Safety Authority, and European Centre for Disease Prevention and Control, 2018). In addition, although *E. coli* is generally harmless to humans and animals, it may constitute a resistant gene pool for transmission of pathogenic bacteria (Marshall et al., 2009), threatening animal and human health. At present, the prevalence of *E. coli* extended-spectrum β-lactamases (ESBLs) is increasing worldwide, which has attracted extensive attention (Pitout and Laupland, 2008). ESBLs can destroy β-lactam antibiotics (Malloy and Campos, 2011), causing these antibiotics to lose their antimicrobial activity and thus making *E. coli* resistant to antibiotics. *E. coli* producing ESBLs in farms may affect public health through environmental pollution and contaminated animal products (Horton et al., 2011). Currently, large-scale pig production generally relies on antibiotics to maintain livestock health and productivity (Robinson et al., 2017). Antibiotic use in pig production may be the major reason for AR in pigs, but it may not solely determine the level of resistance in the pig population. The season, pig type, herd size, and animal exposure may also serve as the factors for emergence and persistence of drug-resistant bacteria.

To understand the factors important for antimicrobial resistance of *E. coli* derived from pigs in large-scale pig farms, a total of 296 strains of *E. coli* isolated and identified from large-scale pig farms were chosen, and their susceptibility to 10 antibiotics were determined by the Kirby-Bauer disk diffusion method in this study. SPSS logistic regression was applied to analyze the effects of multiple factors on the resistance and multi-drug resistance (MDR) of pig-derived *E. coli*, including the season, pig type, sampling position (medication type) and sampling location. This study explored the main factors affecting AR of *E. coli* in pigs and provided a scientific basis for rational use of drugs in large-scale pig farms to control growing bacterial drug resistance.

## Materials and Methods

### Farms

Large-scale pig farms in Shunyi District of Beijing were selected for a visit. The herds were farrow-to-finish herds with at least 800 sows, generating 12,000 heads every year. Herds were visited three times to collect samples for antimicrobial resistance profiling of (nasal and anal) swabs from *E. coli* derived from sick pigs, piglets, sows, nursery pigs, and fattening pigs.

### Isolation and Identification of Pig-Derived *Escherichia coli*

After collection, the swab samples were transported to the laboratory where they were processed within a few hours after arrival (collected sample information shown in Table 1). The swab samples were cultured in trypticase soy broth, followed by overnight incubation at 37°C, while being shaken at 150 rpm. Next, 1 μL loopfuls were used to streak onto MacConkey (MAC) agar plates and incubated for 18–24 h at 36 ± 1°C. Typical brick red to peach red single colonies were selected from MAC and inoculated onto Eosin Methylene Blue (EMB) agar plates (18–24 h, 36 ± 1°C). The isolates were examined according to standard methods of bacteriology and biochemistry. All bacteriological media were acquired from Beijing Land Bridge Technology Co., Ltd (Beijing, China). Isolates were confirmed by 16S rRNA gene sequences, which were 90–100% homologous to *E. coli* by BLAST [Sangon Biotech (Shanghai) Co., Ltd.]. All isolates confirmed as *E. coli* were tested for antibiotic resistance.

**TABLE 1:** Specific sampling volume of a large-scale pig farm in Shunyi District of Beijing (strain).

Type of pig	Spring		Summer				Winter			
	Southern area		Southern area		Northern area		Southern area		Northern area	
	Nasal swab	Anal swab	Nasal swab	Anal swab	Nasal swab	Anal swab	Nasal swab	Anal swab	Nasal swab	Anal swab
Piglet	—	—	5	5	5	5	7	5	5	6
Nursery pig	—	—	5	5	5	5	9	9	—	—
Sow	12	12	6	6	6	6	6	6	6	6
Sick pig	10	—	6	4	5	6	—	—	—	—
Fattening pig	40	—	20	—	10	10	10	—	13	12
Total	74		125				100			

### Test of *Escherichia coli* Susceptibility to Antimicrobial Agents

Susceptibility of *E. coli* isolates was tested on a panel of 10 antimicrobial agents at the 0.5 McF concentration using Kirby-Bauer disk diffusion method. The Clinical and Laboratory Standards Institute (CLSI) standards were followed for inoculum standardization, incubation conditions and internal quality control organisms (Watts et al., 2013). For some antimicrobial agents, susceptibility was tested at two or more concentrations, but resistance was declared only by CLSI-defined resistance breakpoints (Table 2). *E. coli* ATCC25922 was used for quality control.

**TABLE 2:** Interpretation criteria for the diameter of the inhibition zone of *E. coli.*

Class	Antimicrobial	Antibiotic disc (μg/piece)	Interpretation criteria (mm)		
			*S*	*I*	*R*
Chloramphenicol	Florfenicol	30	≥19	15–18	≤14
Semisynthetic penicillin broad-spectrum β-lactam	Amoxicillin	20	≥18	14–17	≤13
Tetracycline	Doxycycline	30	≥16	13–15	≤12
Quinolone	Enrofloxacin	10	≥17	13–16	≤12
Macrolide	Erythromycin	15	≥23	14–22	≤13
Sulfonamide synergist	Trimethoprim	5	≥16	11–15	≤10
Aminoglycoside	Gentamicin	10	≥15	13–14	≤12
Quinolone	Ciprofloxacin	5	≥21	16–20	≤15
Tetracycline	Tetracycline	30	≥15	12–14	≤11
Chloramphenicol	Chloramphenicol	30	≥18	13–17	≤12

### Data Management and Statistical Analysis

Microsoft Excel was used to include all data, and all the data were classified and a library was constructed using Statistical Product and Service Solutions (SPSS, IBM SPSS Statistics version 23) software. Single-factor binary logistic regression was utilized to analyze the effects of four factors, including the season, type of pig, sampling position and sampling location, on resistance of *E. coli*, and the specific assignment instructions were shown in Supplementary Table 1. Based on the odds ratio (OR) value, 95% confidence interval and *P*-value of the OR value were employed to identify possible influencing factors. Then, these influencing factors were added for multivariate binary logistic regression analysis, and significant factors affecting *E. coli* drug resistance were analyzed with *P* < 0.05 (statistically significant) and OR ≠ 1.

By single-factor multiple logistic regression, the influence of four factors on MDR of *E. coli* was analyzed, and specific assignment instructions were listed in Supplementary Table 2. The 95% confidence interval and *P*-value of the OR value helped screen out possible associations with MDR of *E. coli*. Significant factors that affect MDR of *E. coli* were analyzed by adding the selected influencing factors to multi-factor multiple logistic regression analysis based on the magnitude of the OR value with *P* < 0.05 as the significant difference and OR ≠ 1, and then the main way that could affect MDR of *E. coli* was analyzed.

## Results

### Impact Factors of Antimicrobial Resistance to 10 Antibiotics of Pig-Derived *Escherichia coli*

A total of 296 strains identified as *E. coli* were tested for their resistance to 10 antibiotics by drug susceptibility. From Supplementary Table 3, the strains were known highly sensitive to quinolones (enrofloxacin and ciprofloxacin), with the drug resistance rates of only about 4%. In contrast, the tested bacteria showed poor sensitivity to tetracycline drugs including doxycycline, tetracycline and amoxicillin, with the resistance rates being 91.55, 97.64, and 93.24%, respectively. The rate of AR to the banned drug chloramphenicol was 54.39%, which was similar to the special drug florfenicol (68.92%). However, the intermediate sensitivity of the tested bacteria was 0.34% to florfenicol, in contrast with 21.96% to chloramphenicol. The rates of resistance of the tested bacteria to trimethoprim and gentamicin were 68.92 and 15.54%, respectively, and that to macrolide antibiotic erythromycin was 28.72%, which mainly showed an intermediate result (65.54%).

### Single-Factor Binary Logistic Regression Analysis of Pig-Derived *Escherichia coli* Resistance in Different Seasons

**FIGURE 1:**
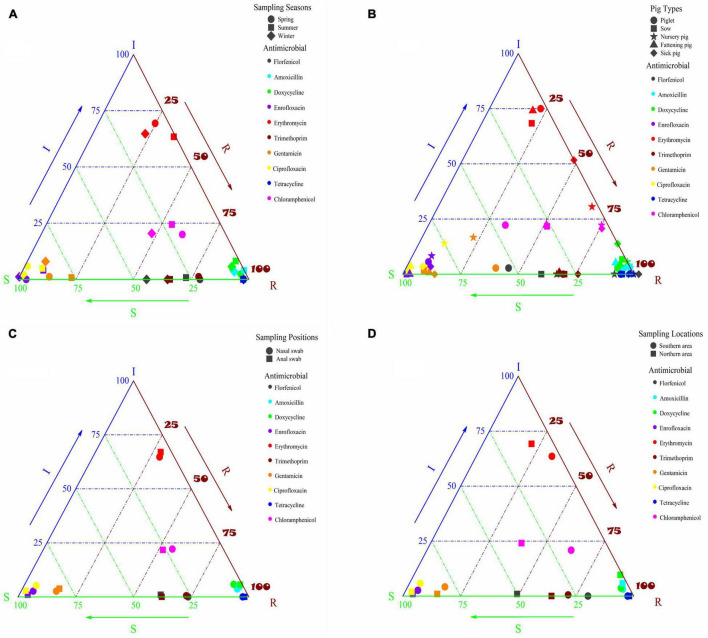
**(A)** Susceptibility of pig-derived *E. coli* to different antibiotics in different seasons; **(B)** susceptibility of *E. coli* derived from different types of pigs to different antibiotics; **(C)** susceptibility of *E. coli* derived from different sampling positions of pigs to different antibiotics; **(D)** susceptibility of *E. coli* derived from pigs in different sampling locations to different antibiotics.

Herein, pig-derived *E. coli* resistance in different seasons was evaluated, and the results were shown in Figure 1A and Supplementary Table 4. The resistance to the 10 antibiotics of the tested bacteria isolated from the samples in spring and summer was higher than in winter. The tested bacteria exhibited high resistance to amoxicillin, tetracycline, and doxycycline despite the season, and their resistance rates were all higher than 90%. The distribution of the tested bacteria’s susceptibility to enrofloxacin and ciprofloxacin was concentrated in spring, summer and winter, and the AR rates were lower than 4% in spring and winter and slightly higher than 8% in summer. The resistance to erythromycin of the tested bacteria was relatively low in spring, summer and winter, with the highest rate of 35.77% in summer and the average rate of 27.74% in these three seasons. It was mainly distributed near the intermediate value, and the results indicated that the tested bacteria might have potential resistance to erythromycin. The tested bacteria were higher resistant to trimethoprim, with an average resistance rate of 69.42%. The drug resistance rate in spring was higher than that in summer and winter, not being an intermediate value. The tested bacteria were more sensitive to gentamicin, with an average resistance rate of 14.55%, and their rates were arranged in descending order in summer (22.76%), in spring (12.94%) and in winter (7.95%).

In order to better understand the influence of seasons on *E. coli* resistance, binary logistic regression analysis was employed. AR of *E. coli* isolated from the samples collected in winter was used as a reference and the results were given in Supplementary Table 5. It could be seen from the table that there was a significant increase in resistance of *E. coli* sampled in spring and summer to all the 10 antibiotics except tetracycline. Compared with *E. coli* sampled in winter, what was sampled in spring and summer showed a significant difference in resistance to florfenicol, and the OR value of florfenicol (2.963) was higher than other antibiotics in spring. Moreover, *E. coli* resistance to gentamicin and erythromycin in summer was significantly different from what was in winter, and the OR values were 3.411 and 1.894, respectively.

### Single-Factor Binary Logistic Regression Analysis of Resistance of *Escherichia coli* Derived From Different Types of Pigs

Resistance of *E. coli* isolated from different types of pigs (sick pigs, piglets, sows, nursery pig and fattening pigs) was evaluated, and the results were shown in Figure 1B and Supplementary Table 6. Overall, the rates of resistance to amoxicillin, tetracycline and doxycycline of the tested bacteria isolated from all types of pigs were high (>86%), and resistance to two quinolones (enrofloxacin and ciprofloxacin) was very low, as the tested bacteria isolated from all pig types showed low AR. The resistance to the two antibiotics of piglets, nursery pigs and sick pigs was higher than that of sows and fattening pigs, with the resistance of nursery pigs shifting from sensitive to intermediate. The resistance to erythromycin of the tested bacteria isolated from piglets, sows and fattening pigs was mainly within the intermediate range, and their resistance rates were low. However, the resistance was higher than that of nursery pigs and sick pigs, especially for that isolated from nursery pigs, for which the resistance rate was 66.67%, and the sensitivity to erythromycin of the tested bacteria began to shift from intermediate to resistant. The tested bacteria isolated from different pig types showed high resistance to trimethoprim, with an average AR rate of 69.88%, and there was no great difference in these pig types. The tested bacteria isolated from piglets and nursery pigs showed high resistance to gentamicin, with the resistance rates of 38.89 and 22.22%, respectively, while the AR rates of the tested bacteria isolated from sows, fattening pigs and sick pigs were low. The tested bacteria isolated from nursery pigs and sick pigs showed high resistance to florfenicol, reaching resistance rates of more than 90%, while the resistance of the tested bacteria isolated from other pig types was relatively low. Similar to florfenicol, the tested bacteria isolated from nursery pigs and sick pigs showed higher resistance to chloramphenicol, with the AR rates reaching 75%. The resistance to chloramphenicol of the tested bacteria isolated from piglets, sows and fattening pigs was low, but the susceptibility mainly fell into the intermediate range, and there was a tendency of changing to resistant.

To understand the influence of pig types on resistance of *E. coli*, binary logistic regression analysis was exploited in this study. AR to 10 antibiotics of *E. coli* isolated from piglets was used as a reference, and all the results were shown in Supplementary Table 7. The AR of *E. coli* in nursery pigs and sick pigs was relatively serious. Compared with piglets, significant differences existed in resistance to florfenicol of *E. coli* from nursery pigs, fattening pigs and sick pigs, and the resistance to florfenicol was very serious in nursery pigs (OR = 12.294). Compared with piglets, resistance to chloramphenicol of *E. coli* from nursery pigs, fattening pigs and sick pigs was also significantly different, and resistance of *E. coli* in nursery pigs was more serious than that in fattening pigs and sick pigs (OR = 6). Similarly, compared with piglets, resistance to erythromycin of *E. coli* from nursery pigs was significantly different (OR = 7). Compared with piglets, there were considerable differences in gentamicin resistance among fattening pigs, sows and sick pigs. By comparing the OR values, it could be found that resistance of piglets to gentamicin was more serious. The results of resistance to florfenicol, chloramphenicol, erythromycin, and gentamicin of *E. coli* isolated from different pig types obtained by binary logistic regression analysis were consistent with those obtained by drug resistance analysis. In short, a significant correlation was presented between pig types and *E. coli* resistance.

### Single-Factor Binary Logistic Regression Analysis of Resistance of *Escherichia coli* Derived From Different Sampling Positions of Pigs

The influence of *E. coli* on antibiotic sensitivity was analyzed according to different sampling sites, and the results were shown in Figure 1C and Supplementary Table 8. The overall resistance to antibiotics other than florfenicol and trimethoprim of the bacteria isolated from nasal swabs and anal swabs basically remained the same. The resistance to amoxicillin, tetracycline and doxycycline of the tested bacteria was divided into two parts, and their resistance rates were all above 90%. The resistance to enrofloxacin and ciprofloxacin was low, despite the resistance of the tested bacteria from nasal swabs was slightly higher than from anal swabs, especially to florfenicol and trimethoprim.

To understand the influence of sampling positions on resistance of *E. coli*, binary logistic regression analysis was utilized. Resistance to 10 antibiotics of *E. coli* isolated from nasal swabs was used as a reference and the results were listed in Supplementary Table 9. There was a significant difference in resistance to florfenicol of *E. coli* from anal and nasal swabs, and the OR values were 0.589, showing that *E. coli* from nasal swabs was more resistant to florfenicol.

### Single-Factor Binary Logistic Regression Analysis of Resistance of Pig-Derived *Escherichia coli* From Different Sampling Locations

The antibiotic sensitivity of *E. coli* from pigs was statistically analyzed according to different sampling locations, and the results were shown in Figure 1D and Supplementary Table 10. The resistance to amoxicillin, tetracycline and doxycycline of the tested bacteria in the southern and northern areas was concentrated (above 90%) except for the tested bacteria’s AR to doxycycline in the northern area (89.42%). It may be attributed to the fact that amoxicillin, tetracycline and doxycycline were used in both the northern and southern areas, which caused high rates of resistance to these three antibiotics. In the northern and southern areas, the tested bacteria were more sensitive to enrofloxacin and ciprofloxacin, and the resistance rates were less than 6%. The tested bacteria in the southern area were slightly more resistant to erythromycin than in the northern area, and they were mainly distributed around the intermediate area, indicating that the tested bacteria in the northern and southern areas are potentially resistant to erythromycin. The tested bacteria had high resistance to trimethoprim and the average resistance rate was 67.89%. The sensitivity to gentamicin of the isolates from the northern and southern areas was relatively high, and the average resistance rate was 15.28%.

The tested bacteria isolated from the southern area had higher resistance to florfenicol and chloramphenicol, reaching the resistance rates of 80.21 and 62.5%, respectively, compared with those from the northern area, with their respective resistance rates of 49.04 and 39.42%. The differences in drug use in the northern and southern areas may be the reason for disparate *E. coli* resistance. Chloramphenicol, banned for veterinary use, possessed a high drug resistance rate for *E. coli* from pigs, similar to florfenicol. The possible reason is that both drugs belong to the class of chloramphenicol and have been affected by the spread of resistance genes.

To understand the influence of sampling locations on resistance of *E. coli*, binary logistic regression analysis was utilized. The resistance to 10 antibiotics of *E. coli* isolated from the northern area was used as a reference and the results were listed in Supplementary Table 11. It could be seen from the table that there was a significant increase in resistance of *E. coli* sampled from the southern area to all the 10 antibiotics except tetracycline. Moreover, resistance to florfenicol, chloramphenicol and erythromycin of *E. coli* from the southern area was significantly different from what was from the northern area, and the OR values were 4.035, 2.561, and 1.82, respectively.

### Multi-Factor Binary Logistic Regression Analysis of Pig-Derived *Escherichia coli* Resistance

**FIGURE 2:**
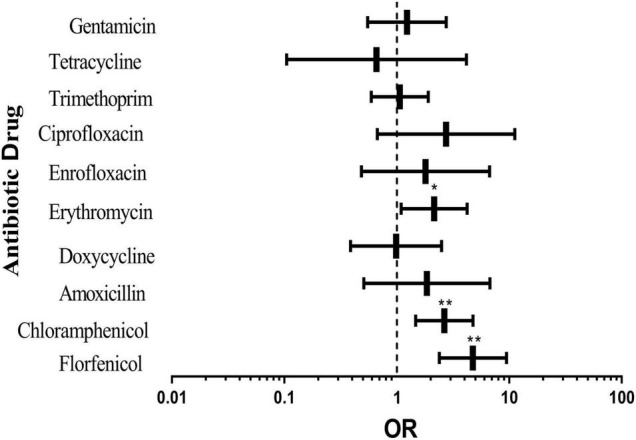
Effects of different sampling locations on pig-derived *E. coli* resistance. The middle box is the OR value representing the odds ratio of *E. coli* resistance to antibiotics in the southern area compared with that in the northern area, the horizontal line is 95% confidence interval, *p < 0.05, and **p < 0.001.

Single-factor binary logistic regression analysis was applied to analyze the relationship between the four factors and pig-derived *E. coli* resistance. There was a significant correlation between AR of *E. coli* and the sampling season, pig type and sampling location. The three factors were introduced into the logistic regression equation for multivariate logistic regression analysis. It could be seen from Figure 2 that resistance to florfenicol, chloramphenicol and erythromycin of the tested bacteria in the southern area was significantly higher than that in the northern area. The results demonstrated that although some antibiotics did not show a tremendous difference, the overall rate of *E. coli* resistance to all antibiotics except tetracycline in the southern area was higher than that in the northern area.

**FIGURE 3:**
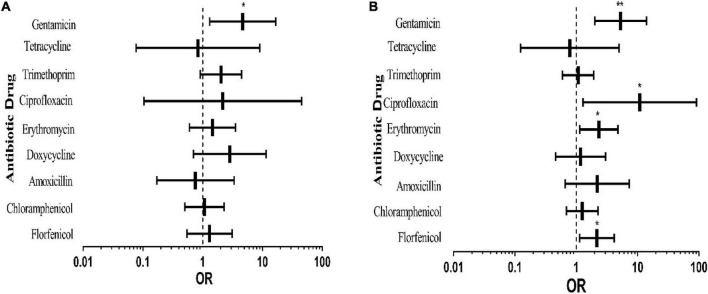
Effects of different sampling seasons on pig-derived *E. coli* resistance. The middle box is the OR value representing the odds ratio of *E. coli* resistance to antibiotics in spring **(A)** and summer **(B)** compared to that in winter, the horizontal line is 95% confidence interval, *p < 0.05, and **p < 0.001.

From Figure 3A, it can be seen that gentamicin resistance of the experimental bacteria isolated in spring was significantly higher than that in winter, and other antibiotics did not show any significant difference. In summer (Figure 3B), resistance to florfenicol, erythromycin, ciprofloxacin and gentamicin of the tested bacteria was significantly higher than that in winter, and the rates of resistance to other antibiotics except tetracycline were higher than that in winter, although there was no substantial difference. The results implied that resistance to antibiotics of the tested bacteria in spring and summer was more serious than that in winter. The causes may be a higher incidence rate of pigs in spring and summer and more serious transmission of pathogens.

**FIGURE 4:**
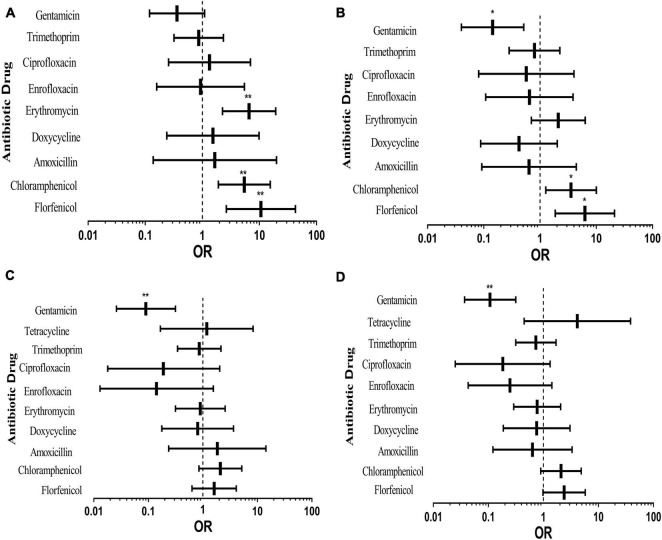
Effects of different pig types on pig-derived *E. coli* resistance. The middle box is the OR value representing the odds ratio of *E. coli* resistance to antibiotics in nursery pigs **(A)**, sick pigs **(B)**, sows **(C)** and fattening pigs **(D)** compared to piglets, the horizontal line is 95% confidence interval, *p < 0.05, and **p < 0.001.

Figure 4 shows the effects of different pig types on drug resistance of *E. coli* from pigs. The rates of resistance to all antibiotics except gentamicin in nursery pigs were higher than that in piglets (Figure 4A). Resistance to erythromycin, chloramphenicol, and florfenicol was vastly different. It can be seen from Figure 4B that the rates of resistance to all antibiotics except erythromycin, chloramphenicol and florfenicol of the experimental bacteria in sick pigs were lower than that in piglets. It was also obvious in Figure 4C that the resistance rate of sows was low, indicating that sows and piglets have similar drug resistance to antibiotics and may possess a vertical transmission risk. It can be seen from Figure 4D that the rates of resistance to all antibiotics except chloramphenicol, florfenicol and tetracycline of the tested bacteria in fattening pigs were lower than that in piglets.

According to the above results, *E. coli* isolated from nursery pigs was more drug-resistant than others. In terms of antibiotics, resistance to gentamicin of piglets was higher than that of other pig types; compared with piglets, *E. coli* from other types of pigs had higher resistance to florfenicol and chloramphenicol. Among them, AR of nursery pigs was the most serious, which may be caused by different growth environments of these pigs. In the process of breeding, pigs are nurtured in different environments at different growth stages, and piglets and nursery pigs grow intensively. Moreover, pigs at this age have low immunity, being prone to diseases, which may lead to *E. coli* AR enhancement. Through multivariate binary logistic regression analysis, it can be seen that antibiotic use, pig growth status and environment have impacts on transmission of *E. coli* drug resistance; meanwhile, there may also be vertical transmission of AR from sows to piglets.

### Analysis of Multiple Factors Influencing Resistance to 10 Antibiotics of Pig-Derived *Escherichia coli* Isolates

A total of 296 isolates of *E. coli* were cultured and tested for susceptibility using a panel of 10 antimicrobials, and the results were listed in Table 3. All the isolates were resistant to the antibiotics, among which 14.53% were resistant to less than 3 antibiotics, and 26.35% were resistant to 6 antibiotics, accounting for the largest proportion. There was even one strain of *E. coli* resistant to all the antibiotics tested.

**TABLE 3:** Multi-drug resistance results of isolated pig-derived *E. coli.*

Multi-drug resistance	Number (%) of drug-resistant strains *n* = 296	Multi-drug resistance	Number (%) of drug-resistant strains *n* = 296
0	0	6	78 (26.35)
1	3 (1.01)	7	46 (15.54)
2	10 (3.38)	8	13 (4.39)
3	30 (10.14)	9	5 (1.69)
4	45 (15.2)	10	1 (0.34)
5	65 (21.96)		

### Single-Factor Multiple Logistic Regression Analysis of Multidrug-Resistant of *Escherichia coli* Derived From Pigs in Different Sampling Seasons

The *E. coli* MDR results were statistically analyzed according to different sampling seasons, as shown in Supplementary Table 12. The analysis focused on resistance to 1–8 antibiotics in spring and winter and to 1–10 antibiotics in summer. One strain of *E. coli* was resistant to all the tested antibiotics. In all the three seasons, 5–6 kinds of antibiotics were predominant in MDR analysis, but the median of MDR in winter was lower than that in spring and summer, indicating that the prevalence of MDR in spring and summer was more serious than that in winter. In all, extreme drug resistance of *E. coli* in summer was the most serious. According to Brower et al. (2017) since several antimicrobials did not follow a normal distribution, a categorical variable describing multidrug resistance was constructed with four levels [0 = susceptible, 1 = singly resistant, 2–4 = moderately multidrug-resistant (MDR), > 4 = extremely MDR].

According to Supplementary Table 13, compared with extreme drug resistance, MDR of *E. coli* in spring was 0.293 times that in winter (CI: 0.143–0.599; *P* < 0.001), revealing that *E. coli* in winter tended to be more multi-drug resistant than that in spring. Meanwhile, MDR of *E. coli* in summer was 0.629 times higher than that in winter. Although there was no significant difference, *E. coli* tended to be more extremely resistant in summer than in winter. In spring and summer, the prevalence of *E. coli* resistance was more serious.

### Single-Factor Multiple Logistic Regression Analysis of Multidrug-Resistant of *Escherichia coli* Derived From Different Types of Pigs

The *E. coli* MDR results were statistically analyzed according to different pig types, as shown in Supplementary Table 14. MDR of sick pigs, fattening pigs, sows and piglets was mainly concentrated in 4–5 antibiotics, while that of nursery pigs was mainly concentrated in 7–8 antibiotics, demonstrating that the prevalence of extreme AR of nursery pigs was more serious.

According to Supplementary Table 15, with extreme drug resistance considered, the MDR odds of *E. coli* from nursery pigs were 0.229 times that of piglets (CI: 0.065–0.804; *P* < 0.05), indicating that *E. coli* from nursery pigs was more likely to obtain extreme drug resistance than piglets. It also showed that the distribution of *E. coli* resistance in piglets and sows was similar. The comparison of the OR values showed that there was little difference in MDR between piglets and sows (OR = 1.013). From the analysis of different pig types, the extreme drug resistance of *E. coli* in nursery pigs was more serious, and there was a possibility of vertical transmission from sows to piglets.

### Single-Factor Multiple Logistic Regression Analysis of Multidrug-Resistant of *Escherichia coli* Derived From Different Sampling Positions of Pigs

*E. coli* MDR was statistically analyzed according to different sampling sites of *E. coli* from pigs, and the results were displayed in Supplementary Table 16. MDR of *E. coli* from nasal swabs and anal swabs was mainly concentrated in 5–6 antibiotics and the results were listed in Supplementary Table 17. Compared with those from anal swabs, *E. coli* from nasal swabs was mostly concentrated in the MDR category, and extreme resistance of *E. coli* from anal swabs was more serious. Meanwhile, there was no significant difference between the two. Therefore, the sampling position was not used as a dependent variable for multi-factor multinomial logistic analysis.

### Single-Factor Multiple Logistic Regression Analysis of Multidrug-Resistant of *Escherichia coli* Derived From Pigs in Different Sampling Locations

MDR of *E. coli* from pigs was analyzed according to different sampling locations, and the results were shown in Supplementary Table 18. MDR in the southern area was mainly concentrated in 5–7 antibiotics, while that in the northern area was mainly concentrated in 3–5 antibiotics, suggesting that the prevalence of extreme drug resistance of *E. coli* was more serious in the southern area.

According to Supplementary Table 19, compared with extreme drug resistance, the MDR odds of *E. coli* in the northern area were 4.851 times higher (CI: 2.831–8.312; *P* < 0.001) than those in the southern area. Resistance of *E. coli* in the southern area was more prone to extreme drug resistance than that in the northern area. Analysis from different sampling locations demonstrated that the prevalence of MDR and resistance to 10 antibiotics of *E. coli* in the southern area was higher than those in the northern area, indicating that the sampling location was an important factor affecting *E. coli* drug resistance.

### Multi-Factor Multiple Logistic Regression Analysis of Multidrug-Resistant to 10 Antibiotics of Pig-Derived *Escherichia coli*

Single-element multiple logistic regression was used to analyze the relationship between the four factors and MDR of *E. coli* from pigs. Among them, the sampling season, pig type and sampling location were significantly associated with MDR of pig-derived *E. coli*. The three factors were introduced into logistic regression equation to carry out multi-factor multiple logistic regression analysis. According to Supplementary Table 20, compared with extreme drug resistance, MDR of *E. coli* in the northern area was 4.113 times that in the southern area (CI: 2.144–7.89; *P* < 0.001), which indicated that *E. coli* in the southern area was more likely to produce extreme drug resistance than that in the northern area. After logistic regression analysis of the three factors at the same time, only the sampling location was significant, revealing that it was the main factor affecting MDR of *E. coli* from pigs. The biggest difference between the two sampling locations was caused by the type of drug used. The use of antibiotics was an important factor affecting extreme AR of *E. coli* from pigs.

### Detection and Analysis of Extended Spectrum β-Lactamases in Isolated Pig-Derived *Escherichia coli*

The expression of extended spectrum β-lactamases (ESBLs) in 85 strains of *E. coli* isolated from pig farms in spring was detected by paper agar amplification. There was only one strain (1.18%) of *E. coli* that can produce ESBLs, and the results were given in Supplementary Table 21.

## Discussion

By exploring the differences in resistance of pig-derived *E. coli* based on types of drugs, pig types, seasons and sampling locations, it is possible to find out the risk factors that trigger bacterial resistance in the pig breeding process and to analyze the factors affecting bacterial resistance, which provides a theoretical basis for mitigating the transmission of drug resistance of *E. coli*.

Comprehensive analysis of AR and MDR distribution of pig-derived *E. coli* revealed that *E. coli* is especially sensitive to quinolones and aminoglycoside drugs, highly resistant to tetracyclines and penicillin, and relatively resistant to sulfonamides and chloramphenicol. The rate of *E. coli* resistance to erythromycin is low, mainly concentrated between susceptibility and resistance, indicating that *E. coli* is potentially resistant to erythromycin. This warns farmers that use of macrolide drugs should be controlled to reduce the risk of *E. coli* resistance to macrolide drugs. Pig-derived *E. coli* is mainly resistant to 5–6 kinds of antibiotics, and the ratio of resistance to more than 3 kinds of drugs is 85.47%, indicating that resistance of *E. coli* in farms is relatively serious. Similar *E. coli* resistance results have been found in the study that tested pig farms in France, Italy, Denmark and Sweden (Burow et al., 2019). A study has also shown that pig-derived *E. coli* is highly resistant to tetracyclines and penicillin and relatively more sensitive to aminoglycoside drugs in pig farms in Thailand (Strom et al., 2017), which is similar to our results. Zhang et al. (2017) studied the resistance trend of *E. coli* in food animals in China from 2008 to 2015 and discovered that pig-derived *E. coli* has high MDR in the country, and the rates of resistance to several earlier drugs such as ampicillin and tetracycline are high (> 80%), suggesting that pig-derived *E. coli* in global pig farms is highly resistant to tetracyclines, penicillin, and sulfonamides, which may be related to long-term, widespread use of these three types of drugs in the pig-feeding industry.

Infections caused by MDR Gram-negative bacteria could be treated by colistin, which has been utilized as the last-resort antimicrobial. Colistin resistance, which is especially associated with mobile genetic elements, such as (mcr) genes, emerged as a major threat to the control of infections caused by Gram-negative bacteria. Dadashi et al. (2021) investigated the global prevalence of mcr-mediated colistin-resistant *E. coli* clinical isolates in Oceania, Africa, America, Europe, and Asia, which were 0.32, 2.27, 5.19, 25.49, and 66.72, respectively. Their research included 79% of the works published between 2014 and 2020 (Dadashi et al., 2021). Shen et al. (2018) investigated 774 non-duplicate MCR-1-positive *E. coli* (MCRPEC) isolates from 774 stool samples collected from 5,159 healthy individuals in 30 provinces and municipalities in 2016, with MCRPEC prevalence ranging from 3.7 to 32.7% (average: 15.0%)—substantially higher than previously reported (Shen et al., 2018).

As human aplastic anemia and certain reproductive/hepatotoxic issues in animals could be induced by chloramphenicol, its use in food, feed, medicine and aquaculture production has been banned for at least 20 years in different parts of the world (Sathya et al., 2020; Wang et al., 2021). Since 2002, China has clearly stipulated that chloramphenicol is prohibited in animal-source foods, as described in Announcement No. 235, “Maximum Residue Limits of Veterinary Drugs in Foods of Animal Origin.” Similarly, concerns about the use of chloramphenicol were expressed and related regulations were set by different countries and regions including the US, Canada, European Union, Australia, Japan, and Brazil, because of bacterial resistance to chloramphenicol and associated clinical toxicity (Barreto et al., 2016). When it is exposed to antimicrobial organisms that are multidrug resistant, its resistance to unrelated drugs will increase. According to the relationship between aminoglycoside and chloramphenicol resistance genes indicated by many previous studies, existence of aminoglycosides and macrolides in lactating piglets may lead to persistence of chloramphenicol resistance in growing pigs (Bischoff et al., 2005; Travis et al., 2006).

Among all types of pigs, nursery pigs and sick pigs had more serious *E. coli* resistance than piglets, fattening pigs and sows, and the epidemic situation of extreme drug resistance of *E. coli* was more serious in nursery pigs. SPSS logistic regression equation was used to analyze the effects of pig types on AR and MDR of pig-derived *E. coli*. It was found that *E. coli* resistance for piglets and nursery pigs in pig farms was more serious, which was similar to the situation in pig farms in Liaoning and other provinces, indicating that most pig farms were facing high *E. coli* AR in piglets and nursery pigs across the country. This may be ascribed to younger ages of piglets and nursery pigs, underdevelopment of their bodies, low immunity and susceptibility to diseases. In the treatment process, resistance of *E. coli* is further enhanced due to the influence of selective pressure. The spread of infectious diseases in pig farms is affected by many factors such as the feeding mode and disease control, herd size, biosecurity and sanitation level (Alvarez-Ordonez et al., 2013; Laanen et al., 2013). These factors may vary between herds and countries and indirectly lead to resistance.

In addition, the feeding mode in pig farms is influential in spreading pathogens and resistant bacteria (Liebana et al., 2013; Tobias et al., 2014). Limited transmission between pig herds reduces the spread of infectious diseases and drug-resistant bacteria, which in turn mitigates bacterial resistance in pigs and ensures healthy growth of pigs. A study has shown that colonization of bacteria in the intestinal tract of newborns is affected by environmental microbiota of mothers and early delivery rooms (Chen et al., 2018). Belloc et al. (2005) found that piglets from sows treated with quinolones after delivery showed quinolone-resistant *E. coli*, while pigs treated with quinolones 2 months after delivery had less quinolone-resistant *E. coli*. It has also been found that sows treated with lincomycin/macrolides have a higher risk of carrying enrofloxacin-resistant bacteria in their piglets, suggesting that development of bacterial resistance is not limited to used antibiotics (Callens et al., 2015). The results of this investigation showed that distribution of *E. coli* resistance in piglets and sows was similar, and there was a possibility of vertical transmission of *E. coli* resistance from sows to piglets.

In terms of sampling locations, multi-drug resistance in the southern area was mainly concentrated in 5–7 antibiotics, while that in the northern area was mainly concentrated in 3–5 antibiotics. SPSS logistic regression equation was employed to analyze the effects of sampling locations on drug resistance and MDR of pig-derived *E. coli*. It was found that compared with the northern area, *E. coli* in the southern area was associated with a significant increase in resistance to all antibiotics except tetracycline, and the epidemic situation of extreme drug resistance of *E. coli* in the southern area was more serious. The results suggested that *E. coli* resistance in the southern part of the country was more serious than the northern part.

In the preliminary investigation of pig farms, it was found that there were differences in the types of drugs used in the two areas. Amoxicillin, doxycycline, florfenicol, and tilmicosin were used in the southern area, while amoxicillin, doxycycline and Yuanlanjing were mainly used in the northern area. The results demonstrated that resistance of *E. coli* to amoxicillin and doxycycline was relatively high in these two areas, indicating that frequent use of these two drugs in pig farms may impose pressure on bacterial selection and lead to AR. It is worth noting that resistance of *E. coli* to florfenicol in the southern area is significantly higher than that in the northern area. By studying drug use in the two areas, it can be found that drug use is significantly related to formation of bacterial resistance. A total ban on certain antibiotics used for growth promotion (tetracyclines, glycopeptides, and macrolides) and for therapeutic purposes (cephalosporins) in the Netherlands revealed a relationship between antimicrobial use and antimicrobial resistance (Speksnijder et al., 2015), resembling the results of this study.

A study has discovered that there is no resistance to cefotaxime of pig-derived *E. coli* in France, Italy, Denmark and Sweden, which is similar to the results of this test, indicating that resistance to the third-generation cephalosporin of *E. coli* obtained from pig farms is low. However, this does not rule out resistance to the third-generation cephalosporin of certain *E. coli* in swine intestinal flora. Therefore, unless cefotaxime-resistant strains are selectively cultured, the actual degree of this low-level resistance cannot be guaranteed. Zhang et al. (2017) found that the rate of resistance to cefotaxime of *E. coli* from chickens and pigs in China increased from 25.60% in 2008 to 46.91% in 2012, and then dropped to 35.08% in 2015. Compared with EU countries and the United States, the overall rate of resistance to the third-generation cephalosporin in pig farms in China is relatively high (Osterberg et al., 2016), implying that new drugs should be used rationally in livestock and poultry farms in China to prolong the usage of new drugs.

## Conclusion

A total of 85 isolated pig-derived *E. coli* in spring were detected, and there was one strain that could produce ESBLs, while 296 strains of identified *E. coli* were tested for resistance to 10 antibiotics by the drug susceptibility method. The rates of resistance to quinolone, amoxicillin and tetracycline, chloramphenicol and sulfonamides, gentamicin and erythromycin of the tested bacteria were, respectively, about 4%, > 90%, between 50 and 70%, 15.54, and 28.72%. According to the results of resistance to 10 antibiotics of 296 pig-derived *E. coli*, the proportion of resistance was 10.14% to 3 antibiotics, 15.2% to 4 antibiotics, 21.96% to 5 antibiotics, 26.35% to 6 antibiotics, 15.54% to 7 antibiotics, 4.39% to 8 antibiotics and 1.69% to 9 antibiotics. There was only one strain (sample 88) of *E. coli* resistant to all tested antibiotics, defining its relatively high resistance.

In this paper, the effects of seasons, pig types, sampling positions (medication type) and sampling locations on drug resistance and MDR of *E. coli* isolated from pigs were analyzed and it was found that there were significant differences in AR and MDR of *E. coli* from pigs. Among these factors, drug types significantly affected AR and MDR of *E. coli* isolated from pigs. In conclusion, the seasonal change, selective drug use and type of pig have impacts on AR of *E. coli* from pigs according to SPSS logistic regression analysis. The experimental comparison results implied that the medication type was the main factor affecting AR of *E. coli*.

## Data Availability Statement

The original contributions presented in the study are included in the article/Supplementary Material, further inquiries can be directed to the corresponding author/s.

## Author Contributions

SY conceived and designed the experiments. QL and YC provided the detection samples, statistical and data analysis in this research. RZ and JW contributed to the reagents and materials. XL prepared the draft of this manuscript. RL and YW collected the samples. AC revised the article and checked the figures. All authors have read and agreed to the published version of the manuscript.

## Conflict of Interest

The authors declare that the research was conducted in the absence of any commercial or financial relationships that could be construed as a potential conflict of interest.

## Publisher’s Note

All claims expressed in this article are solely those of the authors and do not necessarily represent those of their affiliated organizations, or those of the publisher, the editors and the reviewers. Any product that may be evaluated in this article, or claim that may be made by its manufacturer, is not guaranteed or endorsed by the publisher.

## Abbreviations

*E. coli*: *Escherichia coli*
AR: antibiotic resistance
MDR: multi-drug resistance
OR: odds ratio
CLSI: Clinical and Laboratory Standards Institute.
