# Supplementary material for: Effect of Bacterial Resistance of Escherichia coli From Swine in Large-Scale Pig Farms in Beijing
Source: Front Microbiol. 2022 Mar 31;13:820833. doi: 10.3389/fmicb.2022.820833 (PMC9009224; doi:10.3389/fmicb.2022.820833)
Supplement: Supplementary file 1 [file Table_1.DOCX]

**Table SI1** Assignment Description of Binary Logistic Regression Analysis of *E. coli* Resistance

| Factor | Assignment description |
| --- | --- |
| Sampling location | Northern region = 0, Southern region = 1 |
| Type of pig | Piglet = 0, Nursery pig = 1, Fattening pig = 2, Sow = 3, Sick pig = 4 |
| Season | Spring = 0, Summer = 1, Winter = 2 |
| Sampling position | Nasal swab = 0, Anal swab = 1 |
| Resistance | Sensitivity = 0, Resistance =1 |

**Table SI2** Assignment Instructions for Logistic Regression Analysis of *E. coli* Multi-drug Resistance

| Factor | Assignment description |
| --- | --- |
| Sampling location | Northern region = 0, Southern region = 1 |
| Type of pig | Sick pig = 0, Nursery pig = 1, Fattening pig = 2, Sow = 3, Piglet = 4 |
| Season | Spring = 0, Summer = 1, Winter = 2 |
| Sampling position | Nasal swab = 0, Anal swab = 1 |
| Resistance | All sensitive = 0, One-drug resistance = 1, Multi-drug resistance (2-4) = 2, Extreme drug resistance (> 4) = 3 |

**Table SI3** Sensitivity of the Isolated Pig-derived *E. coli* to 10 Antibiotics (n = 296)

| Antimicrobial | Class | Sensitivity (S) | Intermediate (I) | | Resistance (R) |
| --- | --- | --- | --- | --- | --- |
|  |  | Number (%, S/296)  of strains | | Number (%, I/296)  of strains | Number (%, R/296)  of strains |
| Florfenicol | Chloramphenicol | 90 (30.74) | 1 (0.34) | | 205 (68.92) |
| Chloramphenicol |  | 70 (23.65) | 65 (21.96) | | 161 (54.39) |
| Doxycycline | Tetracycline | 8 (2.7) | 17 (5.74) | | 271 (91.55) |
| Tetracycline |  | 7 (2.36) | 0 (0) | | 289 (97.64) |
| Ciprofloxacin | Quinolone | 271 (91.55) | 13 (4.39) | | 12 (4.05) |
| Enrofloxacin |  | 276 (93.24) | 6 (2.03) | | 14 (4.73) |
| Gentamicin | Aminoglycoside | 241 (81.42) | 9 (3.04) | | 46 (15.54) |
| Erythromycin | Macrolide | 17 (5.74) | 194 (65.54) | | 85 (28.72) |
| Trimethoprim | Sulfonamide synergist | 91 (30.74) | 1 (0.34) | | 204 (68.92) |
| Amoxicillin | Semisynthetic penicillin  Broad-spectrum β-lactam | 8 (2.7) | 12 (4.05) | | 276 (93.24) |

**Table SI4** Resistance of Pig-derived *E. coli* to 10 Antibiotics in Different Seasons

| Antimicrobial | Number (%) of strains | | | Average resistance (%) |
| --- | --- | --- | --- | --- |
|  | Spring  (n = 85) | Summer  (n = 123) | Winter  (n = 88) |  |
| Florfenicol | 67 (78.82) | 89 (72.36) | 49 (55.68) | 68.56 |
| Chloramphenicol | 52 (61.18) | 67 (54.47) | 42 (47.73) | 54.46 |
| Doxycycline | 81 (95.29) | 111 (90.24) | 79 (89.77) | 91.77 |
| Tetracycline | 83 (97.65) | 120 (97.56) | 86 (97.73) | 97.65 |
| Ciprofloxacin | 1 (1.18) | 10 (8.13) | 1 (1.14) | 3.48 |
| Enrofloxacin | 3 (3.53) | 11 (8.94) | 0 (0) | 4.16 |
| Gentamicin | 11 (12.94) | 28 (22.76) | 7 (7.95) | 14.55 |
| Erythromycin | 21 (24.71) | 44 (35.77) | 20 (22.73) | 27.74 |
| Trimethoprim | 66 (77.65) | 81 (65.85) | 57 (64.77) | 69.42 |
| Amoxicillin | 77 (90.59) | 118 (95.93) | 81 (92.05) | 92.86 |

**Table SI5** Binary Logistic Regression Analysis of Pig-derived *E. coli* Resistance to 10 Antibiotics in Different Seasons

| Antimicrobial | OR value (95% CI) | |
| --- | --- | --- |
|  | Spring | Summer |
| Florfenicol | 2.963 (1.517-5.784)** | 2.171 (1.216-3.876)* |
| Chloramphenicol | 1.726 (0.943-3.158) | 1.31 (0.757-2.268) |
| Doxycycline | 2.307 (0.683-7.798) | 1.054 (0.424-2.621) |
| Tetracycline | 0.965 (0.133-7.011) | 0.93 (0.152-5.687) |
| Ciprofloxacin | 1.036 (0.064-16.828) | 7.699 (0.967-61.292) |
| Enrofloxacin | — | — |
| Gentamicin | 1.72 (0.634-4.669) | 3.411 (1.415-8.22)* |
| Erythromycin | 1.116 (0.553-2.249) | 1.894 (1.019-3.52)* |
| Trimethoprim | 1.889 (0.965-3.7) | 1.049 (0.59-1.863) |
| Amoxicillin | 2.452 (0.774-7.772) | 1.202 (0.416-3.474) |

Note: The OR value is the odds ratio of *E. coli* resistance to 10 antibiotics in spring and summer compared to winter, 95% CI is the 95% confidence interval, the rate of *E. coli* resistance to enrofloxacin in winter is 0, the OR value cannot be calculated, *p < 0.05, and **p < 0.001.

**Table SI6** Resistance of *E. coli* Derived from Different Pig Types to 10 Antibiotics

| Antimicrobial | Number (%) of strains | | | | | Average resistance (%) |
| --- | --- | --- | --- | --- | --- | --- |
|  | Piglet  (n = 36) | Sow  (n = 60) | Nursery pig  (n = 36) | Fattening pig  (n = 135) | Sick pig  (n = 29) |  |
| Florfenicol | 16 (44.44) | 36 (60) | 33 (91.67) | 91 (67.41) | 29 (100) | 72.70 |
| Chloramphenicol | 12 (33.33) | 31 (51.56) | 27 (75) | 69 (51.11) | 22 (75.86) | 57.39 |
| Doxycycline | 33 (91.67) | 55 (91.67) | 34 (94.44) | 124 (91.85) | 25 (86.21) | 91.17 |
| Tetracycline | 34 (94.44) | 57 (95) | 36 (100) | 133 (98.52) | 29 (100) | 97.59 |
| Ciprofloxacin | 3 (8.33) | 1 (1.67) | 4 (11.11) | 2 (1.48) | 2 (6.9) | 5.90 |
| Enrofloxacin | 3 (8.33) | 1 (1.67) | 3 (8.33) | 4 (2.96) | 3 (10.34) | 6.33 |
| Gentamicin | 14 (38.89) | 5 (8.33) | 8 (22.22) | 15 (11.11) | 4 (13.79) | 18.87 |
| Erythromycin | 8 (22.22) | 13 (21.67) | 24 (66.67) | 26 (19.26) | 14 (48.28) | 35.62 |
| Trimethoprim | 25 (69.44) | 42 (70) | 24 (66.67) | 91 (67.41) | 22 (75.86) | 69.88 |
| Amoxicillin | 34 (94.44) | 58 (96.67) | 35 (97.22) | 121 (89.63) | 28 (96.55) | 94.90 |

**Table SI7** Binary Logistic Regression Analysis of Resistance of *E. coli* Derived from Different Pig Types to 10 Antibiotics

| Antimicrobial | OR value (95% CI) | | | |
| --- | --- | --- | --- | --- |
|  | Nursery pig | Fattening pig | Sow | Sick pig |
| Florfenicol | 12.294 (3.184-47.468)** | 2.676 (1.257-5.7)* | 1.676 (0.728-3.859) | 5.402 (1.806-16.161)* |
| Chloramphenicol | 6 (2.154-16.712)** | 2.3 (1.06-4.99)* | 2.138 (0.906-5.043) | 3.385 (1.277-8.972)* |
| Doxycycline | 1.545 (0.242-9.85) | 1.082 (0.281-4.159) | 1 (0.224-4.459) | 0.545 (0.12-2.48) |
| Tetracycline | — | 3.735 (0.507-27.495) | 1.118 (0.178-7.03) | — |
| Ciprofloxacin | 1.375 (0.285-6.635) | 0.173 (0.028-1.08) | 0.186 (0.019-1.865) | 0.667 (0.104-4.253) |
| Enrofloxacin | 1 (0.188-5.32) | 0.352 (0.075-1.651) | 0.186 (0.019-1.865) | 1.031 (0.194-5.492) |
| Gentamicin | 0.449 (0.16-1.261) | 0.191 (0.08-0.457)** | 0.143 (0.046-0.444)** | 0.262 (0.082-0.835)* |
| Erythromycin | 7 (2.455-19.957)** | 0.883 (0.361-2.164) | 0.968 (0.357-2.624) | 2.333 (0.87-6.58) |
| Trimethoprim | 0.88 (0.326-2.372) | 0.979 (0.439-2.182) | 1.027 (0.418-2.522) | 0.96 (0.351-2.625) |
| Amoxicillin | 2.059 (0.178-23.773) | 0.574 (0.122-2.688) | 1.706 (0.23-12.67) | 0.627 (0.098-4.003) |

Note: The OR value is the odds ratio of *E. coli* resistance to 10 antibiotics in nursery pigs, fattening pigs, sows and sick pigs compared with piglets, and 95% CI is the 95% confidence interval. The rate of *Escherichia coli* resistance to tetracycline in nursery pigs and sick pigs is 100%, so that it is unable to calculate the OR value. *p < 0.05 and **p < 0.001.

**Table SI8** Resistance of *E. coli* Derived from Different Sampling Positions of Pigs to 10 Antibiotics

| Antimicrobial | Number (%) of drug-resistant strains | | Average resistance (%) |
| --- | --- | --- | --- |
|  | Nasal swab (n = 190) | Anal swab (n = 106) |  |
| Florfenicol | 140 (73.68) | 65 (61.32) | 67.50 |
| Chloramphenicol | 106 (55.79) | 55 (51.59) | 53.84 |
| Doxycycline | 172 (90.53) | 99 (93.4) | 91.96 |
| Tetracycline | 185 (97.37) | 104 (98.11) | 97.74 |
| Ciprofloxacin | 10 (5.26) | 2 (1.89) | 3.57 |
| Enrofloxacin | 10 (5.26) | 4 (3.77) | 4.52 |
| Gentamicin | 29 (15.26) | 17 (16.04) | 15.65 |
| Erythromycin | 55 (28.95) | 30 (28.3) | 28.62 |
| Trimethoprim | 138 (72.63) | 66 (62.26) | 67.45 |
| Amoxicillin | 177 (93.16) | 99 (93.4) | 93.28 |

**Table SI9** Binary Logistic Regression Analysis of Resistance of *E. coli* Derived from Different Sampling Positions of Pigs to 10 Antibiotics

| Antimicrobial | OR value (95% CI) |
| --- | --- |
|  | Anal swab |
| Florfenicol | 0.589 (0.354-0.98)* |
| Chloramphenicol | 0.855 (0.531-1.376) |
| Doxycycline | 1.48 (0.597-3.667) |
| Tetracycline | 1.405 (0.268-7.372 ) |
| Ciprofloxacin | 0.346 (0.074-1.61 ) |
| Enrofloxacin | 0.70 (0.216-2.308 ) |
| Gentamicin | 1.06 (0.552-2.036 ) |
| Erythromycin | 0.969 (0.572-1.64 ) |
| Trimethoprim | 0.622 (0.375-1.031 ) |
| Amoxicillin | 1.039 (0.401-2.689 ) |

Note: The OR value is the odds ratio of *E. coli* resistance from anal swabs to 10 antibiotics compared with that from nasal swabs, and 95% CI is the 95% confidence interval. *p < 0.05 and **p < 0.001.

**Table SI10** Resistance of *E. coli* Derived from Pigs in Different Sampling Locations to 10 Antibiotics

| Antimicrobial | Number (%) of drug-resistant strains | | Average resistance (%) |
| --- | --- | --- | --- |
|  | Southern area (n = 192) | Northern area (n = 104) |  |
| Florfenicol | 154 (80.21) | 51 (49.04) | 64.62 |
| Chloramphenicol | 120 (62.5) | 41 (39.42) | 50.96 |
| Doxycycline | 178 (92.71) | 93 (89.42) | 91.07 |
| Tetracycline | 187 (97.4) | 102 (98.08) | 97.74 |
| Ciprofloxacin | 9 (4.69) | 3 (2.89) | 3.79 |
| Enrofloxacin | 10 (5.20) | 4 (3.85) | 4.53 |
| Gentamicin | 31 (16.15) | 15 (14.42) | 15.28 |
| Erythromycin | 63 (32.81) | 22 (21.15) | 26.98 |
| Trimethoprim | 137 (71.35) | 67 (64.42) | 67.89 |
| Amoxicillin | 180 (93.75) | 96 (92.31) | 93.03 |

**Table SI11** Binary Logistic Regression Analysis of Resistance of *E. coli* Derived from Pigs in Different Sampling Locations to 10 Antibiotics

| Antimicrobial | OR value (95% CI) |
| --- | --- |
|  | Southern area |
| Florfenicol | 4.053 (2.402-6.839)** |
| Chloramphenicol | 2.561 (1.569-4.18)** |
| Doxycycline | 1.504 (0.657-3.444) |
| Tetracycline | 0.733 (0.14-3.847) |
| Ciprofloxacin | 1.656 (0.438-6.254) |
| Enrofloxacin | 1.374 (0.42-4.492) |
| Gentamicin | 1.142 (0.585-2.23) |
| Erythromycin | 1.82 (1.041-3.183)* |
| Trimethoprim | 1.376 (0.827-2.288) |
| Amoxicillin | 1.25 (0.494-3.163) |

Note: The OR value is the odds ratio of *E. coli* resistance to 10 antibiotics in the southern area compared with that in the northern area, and 95% CI is the 95% confidence interval. *p < 0.05 and **p < 0.001.

**Table SI12** Multi-drug Resistance of *E. coli* Derived from Pigs in Different Sampling Seasons

| Multi-resistance | Number (%) of drug-resistant strains | | |
| --- | --- | --- | --- |
|  | Spring (n = 85) | Summer (n = 123) | Winter (n = 88) |
| 0 | 0 | 0 | 0 |
| 1 | 0 | 2 (1.63) | 1 (1.14) |
| 2 | 6 (7.06) | 2 (1.63) | 2 (2.27) |
| 3 | 4 (4.71) | 9 (7.32) | 17 (19.32) |
| 4 | 4 (4.71) | 25 (20.33) | 16 (18.18) |
| 5 | 24 (28.24) | 22 (17.89) | 19 (21.59) |
| 6 | 29 (34.12) | 28 (22.76) | 21 (23.86) |
| 7 | 17 (20) | 19 (15.45) | 10 (11.36) |
| 8 | 1 (1.18) | 10 (8.13) | 2 (2.27) |
| 9 | 0 | 5 (4.09) | 0 |
| 10 | 0 | 1 (0.81) | 0 |

**Table SI13** Multi-drug Resistance Analysis of *E. coli* Isolated from a Pig Farm in Spring and Summer (Reference to Winter) Using Multiple Logistic Regression

| Resistance | Season | OR value (95% CI) |
| --- | --- | --- |
| Single-drug resistance | Spring | — |
|  | Summer | 1.224 (0.108-13.83) |
| Multi-drug resistance | Spring | 0.293 (0.143-0.599)** |
|  | Summer | 0.629 (0.353-1.123) |

Note: The OR value takes winter *E. coli* resistance as a reference using the seasonal factor as an independent variable, representing other resistance levels compared with extreme resistance. 95% CI is the 95% confidence interval. There are no sensitive strains in spring, summer and winter, so the OR value cannot be calculated, and there is no comparison of sensitive categories. There is no strain resistant to a single drug in spring, so the OR value cannot be calculated. *p < 0.05 and **p < 0.001.

**Table SI14** Multi-drug Resistance of *E. coli* Derived from Different Types of Pigs

| Multi-drug resistance | Number (%) of drug-resistant strains | | | | |
| --- | --- | --- | --- | --- | --- |
|  | Piglet (n = 36) | Sow (n = 60) | Nursery pig  (n = 36) | Fattening pig  (n = 135) | Sick pig  (n = 29) |
| 0 | 0 | 0 | 0 | 0 | 0 |
| 1 | 2 (5.56) | 1 (1.67) | 0 | 0 | 0 |
| 2 | 0 | 4 (6.67) | 0 | 9 (6.67) | 0 |
| 3 | 3 (8.33) | 9 (15) | 1 (2.78) | 14 (10.37) | 2 (6.9) |
| 4 | 9 (25) | 17 (28.33) | 3 (8.33) | 20 (14.81) | 9 (31.03) |
| 5 | 6 (16.67) | 14 (23.33) | 7 (19.44) | 38 (28.15) | 5 (17.24) |
| 6 | 8 (22.22) | 11 (18.33) | 7 (19.44) | 35 (25.93) | 5 (17.24) |
| 7 | 5 (13.89) | 3 (5) | 10 (27.78) | 16 (11.85) | 4 (13.79) |
| 8 | 2 (5.56) | 0 | 6 (16.67) | 3 (2.22) | 2 (6.9) |
| 9 | 0 | 1 (1.67) | 2 (5.56) | 0 | 2 (6.9) |
| 10 | 1 (2.78) | 0 | 0 | 0 | 0 |

**Table SI15** Multi-drug Resistance Analysis of *E. coli* Derived from Other Types of Pigs (Reference to Piglets) Using Multiple Logistic Regression

| Resistance | Pig type | OR value (95% CI) |
| --- | --- | --- |
| Single-drug resistance | Sick pig | — |
|  | Nursery pig | — |
|  | Fattening pig | — |
|  | Sow | 0.289 (0.025-3.379) |
| Multi-drug resistance | Sick pig | 0.84 (0.309-2.289) |
|  | Nursery pig | 0.229 (0.065-0.804)* |
|  | Fattening pig | 0.737 (0.331-1.641) |
|  | Sow | 1.013 (0.419-2.449) |

Note: The OR value takes piglet *E. coli* resistance as a reference using different pig types as an independent variable, representing other resistance levels compared with extreme resistance. 95% CI is the 95% confidence interval. There are no sensitive strains in all pig types, so the OR value cannot be calculated, and there is no comparison of sensitive categories. There is no strain resistant to single-drug resistance in sick pigs, nursery pigs and fattening pigs, so the OR value cannot be calculated. *p < 0.05 and **p < 0.001.

**Table SI16** Multi-drug Resistance of *E. coli* Derived from Different Sampling Positions of Pigs

| Multi-drug resistance | Number (%) of drug-resistant strains | | Multi-drug resistance | Number (%) of drug-resistant strains | |
| --- | --- | --- | --- | --- | --- |
|  | Nasal swab  (n = 190) | Anal swab  (n = 106) |  | Nasal swab  (n = 190) | Anal swab  (n = 106) |
| 0 | 0 | 0 | 6 | 53 (27.89) | 25 (23.58) |
| 1 | 2 (1.05) | 1 (0.94) | 7 | 30 (15.79) | 16 (15.09) |
| 2 | 9 (4.74) | 1 (0.84) | 8 | 9 (4.74) | 4 (3.77) |
| 3 | 12 (6.32) | 18 (16.98) | 9 | 3 (1.58) | 2 (1.89) |
| 4 | 27 (14.21) | 18 (16.98) | 10 | 1 (0.53) | 0 |
| 5 | 44 (23.16) | 21 (19.81) |  |  |  |

**Table SI17** Multi-drug Resistance Analysis of Pig-derived *E. coli* Isolated from Nasal Swabs (Reference to Anal Swabs) Using Multiple Logistic Regression

| Resistance | Sampling position | OR value (95% CI) |
| --- | --- | --- |
| Single-drug resistance | Nasal swab | 0.971 (0.087-10.901) |
| Multi-drug resistance | Nasal swab | 0.63 (0.376-1.057) |

Note: The OR value takes resistance of *E. coli* isolated from anal swabs as a reference using different sampling apparatus factors as an independent variable, representing other resistance levels compared with extreme resistance. 95% CI is the 95% confidence interval. There are no sensitive strains from anal swabs and nasal swabs, so the OR value cannot be calculated, and there is no comparison of sensitive categories. *p < 0.05 and **p < 0.001.

**Table SI18** Multi-drug Resistance of *E. coli* Derived from Pigs in Different Sampling Locations

| Multi-drug resistance | Number (%) of drug-resistant strains | | Multi-drug resistance | Number (%) of drug-resistant strains | |
| --- | --- | --- | --- | --- | --- |
|  | Southern area  (n = 192) | Northern area  (n = 104) |  | Southern area  (n = 192) | Northern area  (n = 104) |
| 0 | 0 | 0 | 6 | 58 (30.21) | 20 (19.23) |
| 1 | 2 (1.04) | 1 (0.96) | 7 | 40 (20.83) | 6 (5.77) |
| 2 | 8 (4.17) | 2 (1.92) | 8 | 8 (4.17) | 5 (4.81) |
| 3 | 10 (5.21) | 20 (19.23) | 9 | 3 (1.56) | 2 (1.92) |
| 4 | 15 (7.81) | 30 (28.85) | 10 | 1 (0.52) | 0 |
| 5 | 47 (24.48) | 18 (17.31) |  |  |  |

**Table SI19** Multi-drug Resistance Analysis Using the Multiple Logistic Regression Method of *E. coli* Derived from Pigs in the Northern Area (Reference to the Southern Area)

| Resistance | Sampling location | OR value (95% CI) |
| --- | --- | --- |
| Single-drug resistance | Northern area | 1.539 (0.137-17.33) |
| Multi-drug resistance | Northern area | 4.851 (2.831-8.312)** |

Note: The OR value takes resistance of *E. coli* isolated from the southern area as a reference using different sampling locations as an independent variable, representing other resistance levels compared with extreme resistance. 95% CI is the 95% confidence interval. There are no sensitive strains in the northern area and southern area, so the OR value cannot be calculated, and there is no comparison of sensitive categories. *p < 0.05 and **p < 0.001.

**Table SI20** Influencing Factor Analysis of Multi-drug Resistance of Pig-derived *E. coli*

| Resistance | Influencing factor | | OR value (95% CI) |
| --- | --- | --- | --- |
| Single-drug resistance | Sampling season | Spring | — |
|  |  | Summer | 1.677 (0.141-19.961) |
|  | Pig type | Sick pig | — |
|  |  | Nursery pig | — |
|  |  | Fattening pig | — |
|  |  | Sow | 0.435 (0.036-5.229) |
|  | Sampling location | Northern area | 0.928 (0.078-11.028) |
| Multi-drug resistance | Sampling season | Spring | 0.513 (0.211-1.247) |
|  |  | Summer | 0.593 (0.313-1.124) |
|  | Pig type | Sick pig | 1.003 (0.333-3.024) |
|  |  | Nursery pig | 0.295 (0.08-1.084) |
|  |  | Fattening pig | 1.115 (0.455-2.731) |
|  |  | Sow | 1.303 (0.499-3.406) |
|  | Sampling location | Northern area | 4.113 (2.144-7.89)** |

Note: The OR value takes resistance of *E. coli* from piglets, in the southern area and in winter as a reference using different pig types, sampling locations and seasons as independent variables, representing other resistance levels compared with extreme resistance. 95% CI is the 95% confidence interval. There are no sensitive strains in all strains, so the OR value cannot be calculated, and there is no comparison of sensitive categories. There is no strain resistant to single-drug resistance of sick pigs, nursery pigs and fattening pigs in spring, so the OR value cannot be calculated. *p < 0.05 and **p < 0.001.

**Table SI21** Results of ESBLs Produced by Pig-derived *E. coli* in Spring

| ESBL production | Number (%) of strains (n = 85) |
| --- | --- |
| ESBL producible | 1 (1.18) |
| Non ESBL producible | 84 (98.82) |
