# Supplementary material for: Effect of Bacterial Resistance of Escherichia coli From Swine in Large-Scale Pig Farms in Beijing
Source: Front Microbiol. 2022 Mar 31;13:820833. doi: 10.3389/fmicb.2022.820833 (PMC9009224; doi:10.3389/fmicb.2022.820833)
Supplement: Supplementary file 2 [file Table_2.pdf]

Table2 The mean values of isolates 296 for the individual isolates

	number	original number	Florfenicol FF/FON 30ug/piece			Amoxicillin AMX 20ug/piece			Doxycycline DX 30ug/piece			Enrofloxacin ENR 10ug/piece			Erythromycin E 15ug/piece			Trimethoprim(TMP) 5ug/piece			Gentamicin GM 10ug/piece			Ciprofloxacin CIP 5ug/piece			Tetracycline TE 30ug/piece			Chloramphenicol C 30ug/piece			Sensitivity	Intermediate	Resistance
			Sensitivity	Intermediate	Resistance	Sensitivity	Intermediate	Resistance	Sensitivity	Intermediate	Resistance	Sensitivity	Intermediate	Resistance	Sensitivity	Intermediate	Resistance	Sensitivity	Intermediate	Resistance	Sensitivity	Intermediate	Resistance	Sensitivity	Intermediate	Resistance	Sensitivity	Intermediate	Resistance	Sensitivity	Intermediate	Resistance			
			≥19/mm	18–15/mm	≤14/mm	≥18/mm	17–14/mm	≤13/mm	≥16/mm	15–13/mm	≤12/mm	≥17/mm	16–13/mm	≤12/mm	≥23/mm	22–14/mm	≤13/mm	≥16/mm	15–11/mm	≤10/mm	≥15/mm	13–14/mm	≤12/mm	≥21/mm	20–16/mm	≤15/mm	≥15	12——14	≤11	≥18/mm	17–13/mm	≤12/mm			
Spring	1	SG4			6			6			6	21.64				18.12		27.23			24.5			29.13					6			10.5	4	1	5
	2	YN3RB			10			6			12	32			23					9	24			33					6			6	4	0	6
	3	YN19			8.45			6			9.35	26.35				18.7				6			9.4	30.7					7			6	2	1	7
	4	YN29			6			6			6	22.05				14.6		29.25			21.75			31.45					6			6	4	1	5
	5	YN5	22			13.5					9.5	25.83				13.97		26.19			23.5			25.14					7.5	28.7			7	1	2
	6	YN5R			10			6			12	24				19				6	23			30					6			6	3	1	6
	7	YN28			11.05			6			6	24.44					12.66			6	21.35			29.35					6			6	3	0	7
	8	YN27			7.6	20					6	28.65					12.9			6		13.6		27					6			6	3	0	7
	9	YN27R			10			6			10	26					13			6	23			28					6		14.89		4	1	5
	10	YN15			6			6			9.5	28.58				15.74				9	25			23.73					6		14.33		3	2	5
	11	YN34			8.9			6			9.5	22.3				19.85				6	21.75			22.85					6			6	3	1	6
	12	YN34RB			6			6			9	24				17		25			23			28					6			6	4	1	5
	13	YN10	22					6			6	25.92				15.8		25.46			23.5				19.9				6	27.14			5	2	3
	14	YN10GB2			10			6			11	31				22		31			24			35					6			6	4	1	5
	15	YN17	28.85					6			6	39.2					12.25	17.8			24.5					6			6		15.8		4	1	5
	16	YN18			11.3			6			12.85	26.3			23.8					6	22.65			31.3					6		16		4	1	5
	17	YN18RB			11			6			12	25				16				6	21			26					6		14.34		3	2	5
	18	YN40			6			6			10.2	26.55				17.45				6	22.7			24.55					6			6	3	1	6
	19	YN21			10.25			6	16					11.1		18.4				6			7.5		16.15		19.07				13.14		2	3	5
	20	YN16			10.45			6			9.405	28.55				14.3				6		13.65		22.4					6			6	2	2	6
	21	YN16RB			12			6			10	25				19				6	22			22					6			6	3	1	6
	22	YN38			7.95			6			9.6	28.05				13.6				6	24.2			28.6					6		13.66		3	2	5
	23	YN25	18.05					10.3			8.8	26.1			28.8					6			8.95	37.25					6	28.78			5	0	5
	24	YN25GB	20					10			8	25					6			6			6	30					6	23.56			4	0	6
	25	YN12			9.8			6			8.5	23				16.13				6	24			25.71					6			6	3	1	6
	26	YN36			12.3			6			9.05			11.05			13.55			6	24.45			29.1					6			6	2	0	8
	27	YN36RB			7			6			11	31					10			6	20			36					7			6	3	0	7
	28	YN36RB2			8			6			10	32					9			6	19			28					6			6	3	0	7
	29	YN23			8.45			6			8.7	26.6				20.95				6	22.45			21.7					6		15.2		3	2	5
	30	YN24			10.75			6		13.75		27.6			25.4					6	24.55			33.45					6		16.8		4	2	4
	31	YN20	18.8					10.9			9.5	28.2					13.55			6			7.95	37.85					6	27.16			4	0	6
	32	YN4	24			18.5	17				8	25.71				9.77		27.32			21.5			28.06					7.5	27.7			7	1	2
	33	YN4RB	21			20					10	24				19		29			21			25					6	25			7	1	2
	34	YN35	22.95					6			7.95	26.9				14.7				6	22.05			27.05					6		14.75		4	2	4
	35	YN37			8.3			6			8.45	32.7					10.6		14.9		24.95			33.9					6			6	3	1	6
	36	YN30	20.2				16.35				8.7	28.1				18.95		26.95			19.2			22.6					6	26			6	2	2
	37	YN30RB	23					6			6	27				15				6	19			33					6	18.17			5	1	4
	38	YN2			6			6			8	23.04				17.33				6	25			26.57					6			6	3	1	6
	39	YN1			6			6			9	24.45				18.79				6	26			24.78					6			6	3	1	6
	40	YN14			6			6			6	21.2				16.81				6			9.5	22.29					6			6	2	1	7
	41	YN14R			6			6			8	24					6			6	22			32					6			6	3	0	7
	42	YN14RB			12			6			7	35				21				6	20			38					6			6	3	1	6
	43	YN39			7.35			8.4			6.95	39.9					7.55			9.05	24.95			43.3					6			6	3	0	7
	44	YN32			9.35			6	20.2			24.3			25.95					6	23.9			22.3					6			6	5	0	5
	45	YN31			9.75			6			10.4			12.14		16.95				6	25.4				19.15				6		14.5		1	3	6
	46	YN31RB			7			6			10	29				15				7	22			27					6		14.67		3	2	5
	47	YN8			6			6			8	20.65				19.53				6	23			23.83					6		14		3	2	5
	48	YN11			12.5			6			10	24.05				17.05				6	27.5			25.49					6		12.9		3	2	5
	49	YN13			6			6			8	23.26					6			6	22			21.88					6			6	3	0	7
	50	YN33			11.35			6			10.35	33.55				16.75	9.7			8.2	24.46			38.75					6			6	3	1	6
	51	YN33R	23					6			6	26				16				6	21			31					6	30			4	2	4
	52	YN6			6			6			6	21.92				17.39				6	24.5				17.21				6			6	2	2	6
	53	YN9			12			6			11.5	28.77				18.13				6	22.5			29.81					6		16.7		3	2	5
	54	YN9RB			9			6			10	25				21				6	22			26					6		13.6		3	2	5
	55	YN22			8			6			7.5	24.8				20.4		25.9			24.35			27.1					6			6	4	1	5
	56	YN26			7.45			6			9.65	21.5				12.65				6	22.1				20.05				6			6	2	2	6
	57	YN26RB	21				17				9	31				19		26			19			25					6	26			6	2	2
	58	YN26R	23				16				10	30				14		27			20			33					6	26.16			6	2	2
	59	MG1			9			6			12	29				14				6	21			32					6			6	3	1	6
	60	MG2			8			6			7	22				18		25			18			22					6			7	4	1	5
	61	MG4	21					6			12	27				17		31			17			29					7	30.7			6	1	3
	62	MG5	22					6			12	29				14		31			17			32					6	30			6	1	3
	63	MG7	21					6		13		25				15				6	17			26			19.5					6	5	2	3
	64	MG8			6			6			8	24					11			6	16			26					6			6	3	0	7
	65	MG8RB			7			6			10	26				22				6	22			29					6			7	3	1	6
	66	MG9			6			6			12	17				17				6	22			22					7			6	3	1	6
	67	MG9R			7			6			10	25				19				6	25			29					6			6	3	1	6
	68	MG10			8			6			7	20					13			6	17			21					6			6	3	0	7
	69	MG11			6			6			6	20					12			6	17			23					6			7	3	0	7
	70	MG12			7			6			12	21				15				6	16			23					7			6	3	1	6
	71	MN4	20					6			10	18				16				6			9	21					6	24.5			4	1	5
	72	MN6			6			6			9	19				18				6	16			21					6			[illegible]	3	1	6

[illegible]
